# Male breast metastases from nasopharyngeal carcinoma: A case report and literature review

**DOI:** 10.3892/ol.2014.1894

**Published:** 2014-02-20

**Authors:** NING LIANG, JIAN XIE, FENGJUN LIU, DEGUO XU, XINSHUANG YU, YUAN TIAN, MEIJUAN SONG, JIANDONG ZHANG

**Affiliations:** 1 Division of Oncology, Graduate Department, Weifang Medical College, Weifang, Shandong 261031, P.R. China; 2 Division of Oncology, Graduate Department, Shandong University School of Medicine, Jinan, Shandong 250012, P.R. China; 3 Department of Radiation Oncology, Qianfoshan Hospital Affiliated to Shandong University, Jinan, Shandong 250014, P.R. China

**Keywords:** nasopharyngeal carcinoma, male, breast, metastasis

## Abstract

Nasopharyngeal carcinoma (NPC) is known for its high rate of regional lymph node and distant metastasis. However, NPC rarely metastasizes to the breast and, to the best of our knowledge, only four well-documented cases of breast metastasis have previously been reported in the literature, all of which are female. A 49-year-old male was diagnosed with NPC and developed a right breast mass five months later. Breast fine needle aspiration confirmed an abundance of metastatic squamous cells within the thickened tissue. The current study presents the first male case of breast metastases from NPC to broaden the clinical database.

## Introduction

Although the occurrence of nasopharyngeal carcinoma (NPC) is rare in the Western hemisphere, it is a common malignancy in Southern China (1,2). Compared with other head and neck squamous cell carcinomas, NPC is associated with the highest rates of regional lymph node and distant metastasis (3). The most common site of metastasis is the cervical lymph nodes (4), and the majority of NPCs have already metastasized at the point of diagnosis. Radiotherapy is considered to be the predominant regimen for the treatment of almost all NPC cases, with a five-year overall survival (OS) rate of 87–96% for stage I-II and 67–77% for stage III-IVB (5). However, local recurrence remains the major cause of treatment failure following conventional radiotherapy, and distant metastasis is often observed following conventional treatment. Although NPC is known for its high rate of metastasis, metastasis to the breast is extremely rarely and, to the best of our knowledge, only four well-documented cases have been reported in the literature to date. The four female cases include two cases reported in 1991, one case reported in 2004, which was one of 15 cases of extramammary breast metastases, and a case that was reported in 2007 (6–8). Thus far, no male cases of breast metastasis from NPC have been reported. Therefore, to the best of our knowledge, the current study reports the first case of male breast metastasis from NPC.

## Case report

**Figure 1:**
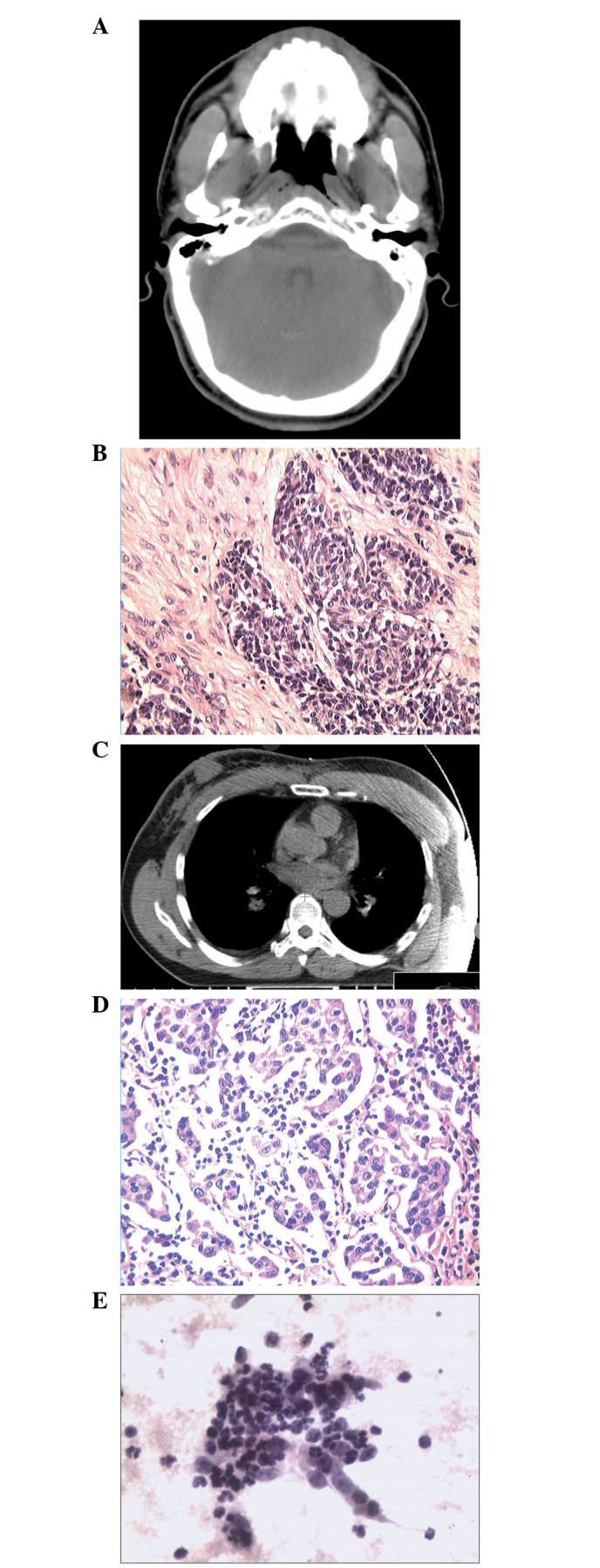
(A) Computed tomography (CT) scan of the head and neck showed thickening of the soft tissues of the right wall of the nasopharynx. (B) Histopathological examination of the nasopharynx revealed undifferentiatd squamous cells. (H&E staining; magnification, ×10). (C) CT scan of the chest showed a locally thickened wall of the right chest. (D) Histopathological examination of the right axillary lymph node specimens revealed a number of poorly differentiated metastatic squamous cells. (H&E staining; magnification, ×40)(E) Breast fine needle aspiration revealed metastatic squamous cells.

A 49-year-old male presented to Qianfoshan hospital affiliated to Shandong University (Jinan, China) in August 2009 with a lump in the right upper neck region, which had been growing for four months. Physical examination identified multiple circular lumps, which were palpable on the right upper third of the neck on the sternocleidomastoid (facies medialis). The largest lump, measuring 5×4 cm, had a hard texture and was adhered to the surrounding tissue. In the right supraclavicular fossa, multiple active bilateral nodes with a hard texture were identified. Fiberoptic nasopharyngoscopy revealed lifting the right side of the tonsillar crypts and the surface was rough (fiberoptic instruments: OTVS7PRO, CLVS4OPRO and OTV-StPro; Laryngoscopic lens, ENF-VQ 2440288, Olympus Corporation, Tokyo, Japan). Examination of the head and neck by computed tomography (CT) showed thickening of the soft tissues of the right wall of the nasopharynx (Fig. 1A) and bilateral cervical lymphadenopathy with a maximum node size of ~2.9×1.3 cm. Histopathological examination [hemotoxylin and eosin (H&E), Beijing Solarbio Science & Technology Co., Ltd., Beijing, China] of the nasopharynx specimen, viewed under a microscope (IX71, Olympus Corporation) confirmed the diagnosis of undifferentiated squamous cell carcinoma (Fig. 1B) at stage T3N3M0 (9). The patient provided written informed consent.

In the present case. the treatment strategy for the patient involved 3D-conformal radiotherapy of the nasopharynx and cervical lymph nodes (total dose, 70 Gy) concurrent with three cycles of docetaxel/nedaplatin chemotherapy. However, in April 2011, the patient presented with multiple lymph nodes that were tumefied in the two sides of the axillary, generally without fever or pain, although pain was experienced during heavy manual labor. Five months later, the patient identified a mass developing in the right breast. Physical examination confirmed the mass in the right breast and revealed lymphadenopathy on two sides of the axillary. Ultrasonic examination showed thick gland-like tissues behind the right nipple. Chest CT revealed that local tissues of the right chest wall were thickened (Fig. 1C) and also revealed multiple tumid lymph nodes on two sides of the axillary. Histopathological examination (H&E staining) of the right axillary lymph nodes revealed poorly differentiated metastatic squamous carcinoma (Fig. 1D) and breast fine needle aspiration confirmed an abundance of metastatic squamous cells within the thickened tissue (Fig. 1E). In addition, immunohistochemical analysis revealed the apparent expression of cytokeratin (CK) 5/6(+), CK7(−), CK20(−), estrogen receptor(−), thyroid transcription factor 1(−), gross cystic disease fluid protein-15(−) and villin(−). In November 2011, the patient was administered a total dose of 50 Gy palliative radiotherapy to the right axillary and breast in combination with docetaxel plus 5-fluorouracil (FU) and cisplatin chemotherapy for four cycles. In May 2012, the neck and chest CT examination revealed no increase of lymphadenopathy on the two sides of the axillary compared with the previous CT examinations.

## Discussion

NPC is a malignancy endemic to South China with an annual incidence of between 10 and 50 per 100,000 individuals (1), with a tendency to occur in young and middle-aged adults. In contrast to other head and neck tumors, NPC is curable despite regional lymph node spread. Although histologically the common subtypes of NPC are non-keratinizing, keratinizing and undifferentiated, the majority of NPC are undifferentiated. Similar to other head and neck squamous cell carcinomas, NPC is known for its high rate of lymph node metastasis. Due to its presentation below the nasopharyngeal mucosa, which is rich in lymphatics, a lymphatic network subsequently results in lymphatic drainage to the cervical lymph nodes. Therefore, the appearance of cervical lymph node metastases in early NPC is common.

NPC is notably sensitive to chemotherapy and radiotherapy, however, due to anatomical restrictions and radiosensitivity, definitive radiotherapy is regarded as the standard treatment despite the poor response of locoregionally advanced NPC (5). In a previous intergroup 0099 study, concurrent chemoradiotherapy plus adjuvant chemotherapy achieved a 31% increase in three-year OS (10), thus, the authors considered this regimen to be the standard care for advanced NPC. Recently, a phase three multicenter randomized controlled trial revealed that adjuvant cisplatin and FU chemotherapy did not significantly improve failure-free survival in locoregionally advanced NPC following concurrent chemoradiotherapy (11). This result lead the authors to conclude that adjuvant chemotherapy must not be administered following concurrent chemotherapy in patients with locally advanced NPC. Lymphatic metastasis to the cervical lymph nodes is most commonly observed and is a major factor associated with prognosis and treatment outcomes (5). Considering the predominance of cervical lymph node metastasis in NPC, irradiation is commonly administered to the entire neck region in an attempt to control regional recurrence. However, despite the administration of conventional radiotherapy to the irradiation field, including the common sites of local recurrence (such as the clivus and ethmoid), the incidence of distant metastasis following treatment is high. Furthermore, retrospective analysis of the clinical outcomes and patterns of treatment failure for NPC with distant metastasis following intensity-modulated radiation therapy remains challenging (12). Nevertheless, distant metastasis to the breast from NPC is rare, as breast metastasis usually occurs as a result of a disseminated disease that is indicated by extramammary neoplasms (13). In the current study, two years following the initial treatment the patient received definitive treatment due to the first presentation of axillary lymph node metastasis, which was followed a few months later by tumefaction of the right breast. The tumefaction indicated that the NPC had metastasized to the breast, perhaps as a result of the axillary lymph node metastasis. Although a search of the English literature using PubMed retrieved four previously reported cases of breast metastasis from NPC, all four cases were female and no male cases had been reported. Therefore, the current study established that NPC may also metastasize to the breast in males and highlights the importance of breast examination in the follow-up of NPC patients.

In patients with recurrent or distant metastatic NPC, platinum-based chemotherapy is considered to be the preferred regimen for first-line treatment (14). However, no standard strategy has been established for local recurrence or metastasis. To prolong the survival time of such patients, a series of studies have been performed to identify effective and tolerable regimens. In a previous phase II study of gemcitabine-vinorelbine chemotherapy in metastatic NPC patients that were pretreated with platinum-based chemotherapy (15), the overall response rate was 37.7%, with an OS of 14.1 months and progression-free survival (PFS) of 5.2 months. In addition, the hematologic and non-hematologic toxicities were mild. Therefore, the authors concluded that this strategy is effective and safe for the treatment of such patients. In an additional phase II study of ifosfamide in the treatment of recurrent NPC patients, 5-FU and leucovorin was used as the second-line regimen. The one-year survival rate of the patients was 51% and PFS was 6.5 months, in addition, none of the patients developed grade 3/4 toxicities. In the current report, nasopharyngeal examination of the patient did not reveal local recurrence, but did establish distant metastasis. Therefore, the patient was administrated docetaxel, cisplatin, 5-FU and leucovorin combined with radiotherapy to control the distant metastasis. Following radiotherapy and four cycles of chemotherapy, the condition was evaluated and showed complete remission. Although docetaxel plus platinum was used as the first-line regimen, a docetaxel plus cisplatin combined 5-FU and leucovorin regimen with concurrent radiotherapy achieved improved local control. Compared with previous strategies, this treatment may be effective as a second-line regimen to treat recurrent or distant metastasis. Epidermal growth factor receptor (EGFR) overexpression has been identified in ~85% NPCs, however, a previous phase II trial (16) administered erlotinib, which acts on EGFR, as a maintenance treatment in NPC patients with recurrent and/or metastatic disease following a gemcitabine-platinum regimen and revealed that the treatment was not effective as a maintenance or second-line therapy following chemotherapy. Further studies are required to identify whether, as well as which, molecular targeted agents and strategies are effective for recurrent or metastatic NPC.

In conclusion, the current report confirmed that NPC may also metastasize to the breast in males. In addition, the high rate of NPC metastasis demonstrated an urgent requirement for identifying effective and safe strategies.
